# Toward a Sensible Single-antigen Bead Cutoff Based on Kidney Graft Survival

**DOI:** 10.1097/TP.0000000000002357

**Published:** 2019-03-22

**Authors:** Bram W. Wisse, Elena G. Kamburova, Irma Joosten, Wil A. Allebes, Arnold van der Meer, Luuk B. Hilbrands, Marije C. Baas, Eric Spierings, Cornelis E. Hack, Franka E. van Reekum, Arjan D. van Zuilen, Marianne C. Verhaar, Michiel L. Bots, Adriaan C.A.D. Drop, Loes Plaisier, Marc A.J. Seelen, Jan Stephan Sanders, Bouke G. Hepkema, Annechien J.A. Lambeck, Laura B. Bungener, Caroline Roozendaal, Marcel G.J. Tilanus, Christina E. Voorter, Lotte Wieten, Elly M. van Duijnhoven, Mariëlle A.C.J. Gelens, Maarten H.L. Christiaans, Frans J. van Ittersum, Shaikh A. Nurmohamed, Neubury M. Lardy, Wendy Swelsen, Karlijn A.M.I. van der Pant, Neelke C. van der Weerd, Ineke J.M. ten Berge, Frederike J. Bemelman, Andries J. Hoitsma, Paul J.M. van der Boog, Johan W. de Fijter, Michiel G.H. Betjes, Sebastiaan Heidt, Dave L. Roelen, Frans H. Claas, Henny G. Otten

**Affiliations:** 1 Laboratory of Translational Immunology, University Medical Center Utrecht, Utrecht, The Netherlands.; 2 Radboud University Medical Center, Radboud Institute for Molecular Life Sciences, Laboratory Medicine, Laboratory of Medical Immunology, Nijmegen, The Netherlands.; 3 Radboud University Medical Center, Radboud Institute for Health Sciences, Department of Nephrology, Nijmegen, The Netherlands.; 4 Department of Nephrology and Hypertension, University Medical Center Utrecht, Utrecht, The Netherlands.; 5 Julius Center for Health Sciences and Primary Care, University Medical Center Utrecht, Utrecht, The Netherlands.; 6 Department of Nephrology, University of Groningen, University Medical Center Groningen, Groningen, The Netherlands.; 7 Department of Laboratory Medicine, University of Groningen, University Medical Center Groningen, Groningen, The Netherlands.; 8 Department of Transplantation Immunology, Tissue Typing Laboratory, Maastricht University Medical Center, Maastricht, The Netherlands.; 9 Division of Nephrology, Department of Internal Medicine, Maastricht University Medical Center, Maastricht, The Netherlands.; 10 Amsterdam University Medical Center, Vrije Universiteit Amsterdam, Department of Nephrology, Amsterdam Cardiovascular Sciences, Amsterdam, The Netherlands.; 11 Department of Immunogenetics, Sanquin Diagnostic Services, Amsterdam, The Netherlands.; 12 Amsterdam University Medical Center, University of Amsterdam, Department of Internal Medicine, Renal Transplant Unit, Amsterdam, The Netherlands.; 13 Dutch Organ Transplant Registry (NOTR), Dutch Transplant Foundation (NTS), Leiden, The Netherlands.; 14 Department of Nephrology, Leiden University Medical Center, Leiden, The Netherlands.; 15 Department of Internal Medicine, Nephrology, Erasmus MC, Rotterdam, Department of Nephrology, Rotterdam, The Netherlands.; 16 Department of Immunohematology and Blood Transfusion, Leiden University Medical Center, Leiden, The Netherlands.

## Abstract

**Background.:**

There is no consensus in the literature on the interpretation of single-antigen bead positive for a specific HLA antibody.

**Methods.:**

To inform the debate, we studied the relationship between various single-antigen bead positivity algorithms and the impact of resulting donor-specific HLA antibody (DSA) positivity on long-term kidney graft survival in 3237 deceased-donor transplants.

**Results.:**

First, we showed that the interassay variability can be greatly reduced when working with signal-to-background ratios instead of absolute median fluorescence intensities (MFIs). Next, we determined pretransplant DSA using various MFI cutoffs, signal-to-background ratios, and combinations thereof. The impact of the various cutoffs was studied by comparing the graft survival between the DSA-positive and DSA-negative groups. We did not observe a strong impact of various cutoff levels on 10-year graft survival. A stronger relationship between the cutoff level and 1-year graft survival for DSA-positive transplants was found when using signal-to-background ratios, most pronounced for the bead of the same HLA locus with lowest MFI taken as background.

**Conclusions.:**

With respect to pretransplant risk stratification, we propose a signal-to-background ratio-6 (using the bead of the same HLA-locus with lowest MFI as background) cutoff of 15 combined with an MFI cutoff of 500, resulting in 8% and 21% lower 1- and 10-year graft survivals, respectively, for 8% DSA-positive transplants.

## Abstract 2

Supplemental Digital Content is available in the text.

The complement-dependent cytotoxicity crossmatch (CDC-XM) assays and the much more sensitive Luminex technology are commonly used for the detection of anti-HLA antibodies. The presence of donor-specific anti-HLA antibodies (DSA) detected by the CDC-XM is considered a contraindication for transplant. It is widely known that single-antigen bead (SAB) assays can detect DSAs when the CDC-XM result is negative. The clinical relevance of the antibodies detected by the much more sensitive SAB assays is less clear, however,^1^ and their presence is generally considered to be a risk factor rather than a contraindication.^2^

In the Dutch PROCARE Consortium study, the impact of SAB-detected DSA on graft survival was determined for all kidney transplants performed between 1995 and 2006, for which pretransplant serum was available.^3^ The impact was most pronounced in the 3237 deceased-donor transplants, where the 1- and 10-year graft survivals were 5% and 16%, respectively, lower in patients with SAB-detected DSA in pretransplant sera (N = 430, 13% DSA positive).

In the assessment of SAB bead positivity, initially, the manufacturer’s instructions were followed. In the literature, there is no consensus, however, on the interpretation of these SAB measurements, and there is disagreement on the cutoff value for specific antibodies. This is partly due to the high interassay and interlaboratory variability of the SAB measurements.^4^ Second, and more importantly, contrary to expectations of many, the fluorescence intensity has only a weak relationship with titer of the specific antibody in the tested serum^1,5^ and is influenced by many other factors other than the amount of specific antibody bound to the bead.

We had the opportunity to investigate the relationship between the fluorescence levels measured in pretransplant sera on the one hand, and clinical relevance as expressed by graft survival on the other hand, for a large cohort of CDC-XM–negative kidney transplants performed in the Netherlands between 1995 and 2006. All SAB measurements were performed in 1 central laboratory, using the same machine, bead lot, and by a team of 2 highly trained technicians, keeping the assay variability at a minimum. We agree with recent publications stressing that the interpretation of SAB measurements needs to be made in the context of the patient’s specifics, his/her history, and other available measurements.^6^ The findings of the current rather mechanistic investigation will therefore be informative for general risk stratification rather than for risk assessment of an individual patient.

## MATERIALS AND METHODS

### Interlaboratory Variability

To assess the interlaboratory variability, we have included 12 Eurotransplant Reference Laboratory External Proficiency Testing sera evaluated in 2016 with HLA class I and class II SAB by HLA laboratories in the Eurotransplant region. Of the 12 reference sera, 11 were positive for HLA class I antibodies and 8 for HLA class II antibodies. Here, we have included the measurements of 3 laboratories in the Netherlands using the same SAB kit manufacturer as for our multicenter study.

### Patients, Sera, and Clinical Data

This multicenter study included all 6097 kidney transplants performed between January 1995 and December 2005 in all Dutch transplant centers. In all cases, the T cell and/or unseparated CDC-XM with current and historic peak sera were negative. Historic cytotoxic HLA antibodies were assigned as unacceptable for allocation within the Eurotransplant region. Bead assay–defined DSA were not considered (as risk factor) in the matching procedure at that time, nor had these DSA influence on immunosuppressive treatment. Informed consent for data collection and use of leftover sera was obtained from all subjects. Patients and donors investigated were predominantly white. The use of sera and experimental protocols was approved by the research ethics committee for Biobanks and the medical ethics committee of the University Medical Center Utrecht. Moreover, this study was performed in accordance with the FEDERA code of conduct.

We obtained baseline and clinical follow-up transplant data from the Netherlands Organ Transplant Registry, which was over 95% complete at the time of this study. Clinical follow-up was recorded at 3 and 12 months, and yearly thereafter for at least 10 years after transplant. The primary endpoint of the study is graft failure, defined as loss of kidney function when the patient returns to dialysis or receives a retransplant. In the analysis of death-censored graft failure, recipients who died with a functioning graft were censored at the time of death.

Pretransplant patient sera could be collected from 4787 (78%) transplants of 4585 patients (some patients underwent more than 1 transplant). Seventeen transplants were lost to follow-up (Netherlands Organ Transplant Registry), and 46 transplants were excluded because the kidney failed during surgery or shortly thereafter due to technical nonimmunological problems. Four thousand seven hundred twenty-four transplants were included in the analysis, of which 3237 were performed with grafts from deceased donors.

### Detection and Definition of Donor-specific Anti-HLA Antibodies

The presence of HLA antibodies in the pretransplant sera, used for pretransplant crossmatch, was assessed retrospectively in 1 central laboratory as described previously.^7^ Four thousand one hundred eighty-three (89%) of 4724 of the sera were taken within 3 months pretransplant, 377 (8%) of 4724 were taken 3 to 6 moths pretransplant, and only 164 (3%) of 4724 were taken 6 to 12 months pretransplant. In brief, sera were first tested for the presence of HLA class I and class II antibodies using Lifecodes LifeScreen Deluxe (Immucor Transplant Diagnostics, Stamford, CT). Subsequently, the sera positive for HLA class I and/or class II were analyzed using Lifecodes SAB assay class I and/or II kits (Immucor Transplant Diagnostics) to determine the exact specificity of the HLA antibodies. The LABScan 100 flow analyzer (One Lambda, Canoga Park, CA) was used for data acquisition. Bead positivity assignment, the subject of this study, was evaluated for a range of median fluorescence intensities (MFIs) (median and 5%-trimmed mean), signal-to-background ratio (STBR) cutoffs (Table 1), and combinations thereof. The presence of SAB-DSA was determined by comparing the SAB-HLA-A/B/DR/DQB antibody specificities on serological level with the split level HLA-A/B/DR/DQB typing of the donor.

**TABLE 1.**
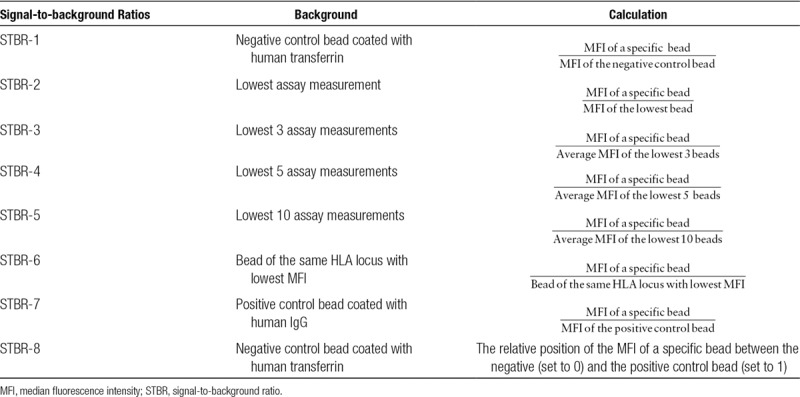
Description of the various signal-to-background ratios with the background used for the calculation

### Statistical Analysis

Death-censored graft survival was assessed using the adjusted Kaplan-Meier estimator based on inverse probability weighting.^8^ The following covariates were considered for adjustment: recipient and donor age, recipient and donor sex, year of transplant, type of donor, cold ischemia time, retransplant, graft function, use of IL-2 receptor blocker, number of HLA-A/B/DR mismatches, transplant, and highest percent PRA.^3^ We adjusted for recipient age (quadratic) and donor age (quadratic), donor type (living or deceased; for the total cohort only), cold ischemia time (for donation after brain death and donation after cardiac death), and induction therapy with IL-2 receptor blocker. Two hundred twenty-six missing cold ischemia times were imputed using Markov chain Monte Carlo single imputation; no additional values were missing. Statistical analyses were performed with R (version 3.3.2) and SAS (version 9.4; SAS Institute, Cary, NC) software.

## RESULTS

### Stabilizing the Fluorescence Measurements

We investigated the direct relationship between fluorescence measurements in Luminex SAB assays and clinical relevance as expressed by graft survival. The fluorescence intensity measured per bead type per sample is highly variable, and in addition, a few beads of the same type from a previous sample can accidentally be carried over by the measuring device. To stabilize the signal and mitigate the effect of beads carried over, typically minimally 60 fluorescence measurements are obtained per bead type per sample and subsequently summarized as either the median or 5% trimmed mean of those measurements. Both the 5% trimmed mean and median values are abbreviated as MFI from the literature; we know also that these MFI values can be highly variable, with studies reporting a coefficient of variation (standard deviation of repeated measurements divided by the average value) of 65% between laboratories.^4^ From personal experience, we know that repeated SAB measurements can show a systematic and seemingly proportional bias between Luminex machines, lots, and test days. A proportional bias can be mitigated by the employment of an STBR (Table 1).

To assess whether interassay variability can be reduced by working with STBRs, we have determined the variability of MFI measurements and compared that with the variability of various STBRs for a set of 12 External Proficiency Testing sera evaluated in 3 laboratories in the Netherlands. Only 1 of these 3 laboratories could recover 5% trimmed mean values in addition to the MFIs. Even though the trimmed mean values tend to be less variable than their median counterparts in our experience (data not shown), we were unable to evaluate this here. In the remainder of this article, MFI will therefore refer to median fluorescence intensities, unless explicitly stated otherwise. In addition, 1 laboratory could not provide the measurements of the negative and positive control beads, which resulted in the absence of these controls as background in the current evaluation of interassay variability. The results of the interlaboratory comparison are given in Table 2 for HLA class I SAB and in Table 3 for HLA class II.

**TABLE 2.**
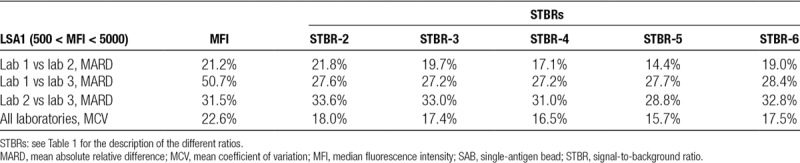
MARD in HLA class I SAB measurements (MFI and various STBR) between laboratories, together with the MCV for all laboratories, in the critical range of average MFI between 500 and 5000

**TABLE 3.**
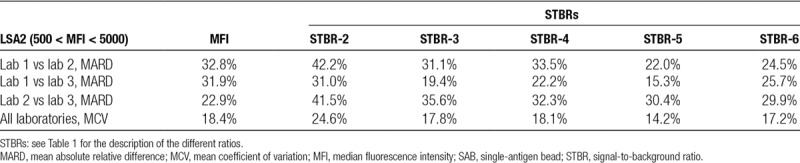
MARD in HLA class II SAB measurements (MFI and various STBRs) between laboratories, together with the MCV for all laboratories, in the critical range of average MFI between 500 and 5000

**FIGURE 1.**
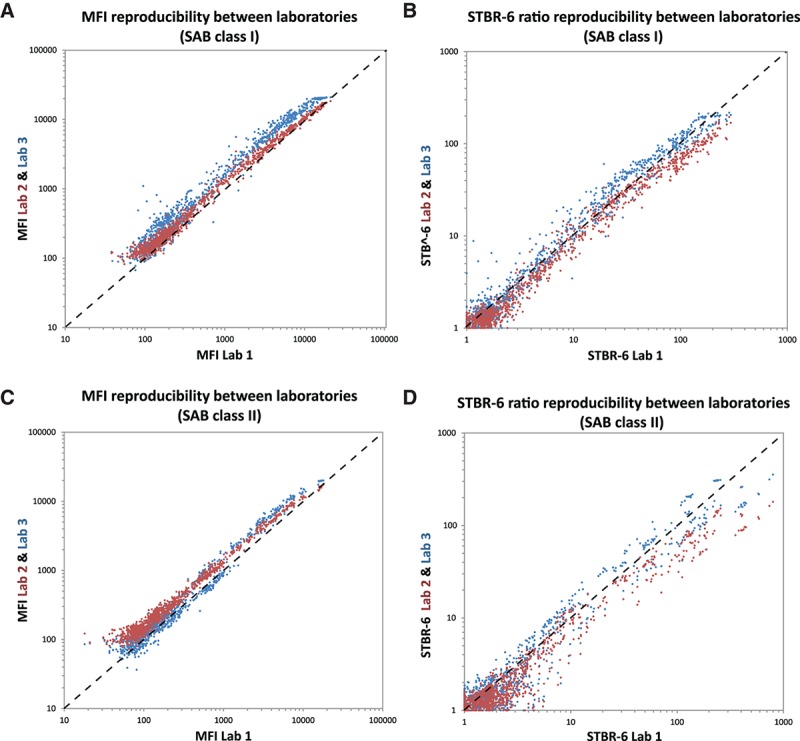
A, SAB class I MFIs of lab 1 plotted against those of lab 2 (red) and 3 (blue). B, SAB class I STBR-6 values using bead of the same HLA locus with lowest MFI as background of lab 1 plotted against those of lab 2 (red) and 3 (blue). C, SAB class II MFIs of lab 1 plotted against those of lab 2 (red) and 3 (blue). D, SAB class II STBR-6 values of lab 1 plotted against those of lab 2 (red) and lab 3 (blue). MFI, median fluorescence intensity; SAB, single-antigen bead; STBR, signal-to-background ratio.

We calculated the mean absolute relative difference (MARD) using the absolute difference in MFI for each HLA bead between lab 1 and lab 2 divided by the average MFI between lab 1 and lab 2. Simplified example: the MARD of +25 and −23 is 24. For SAB class I beads, we found a MARD in MFI measurement between 2 laboratories ranging from 21% (lab 1 vs lab 2) to 51% (lab 1 vs lab 3) in the critical range of average MFIs between 500 and 5000. For the ratios, the MARD between the 3 laboratories was consistently smaller, with values between 14% and 34%. Also, the mean coefficient of variation (MCV) was lower for the STBRs (values between 16% and 18%) than for the raw MFI (23%). Within the group of STBRs, generally more stable results (the smallest MARD and MCV) were obtained when more of the lowest ranked beads were used to derive a background fluorescence. Normalizing by the MFI of the bead of the same HLA locus with lowest MFI (which can be loosely interpreted as the bead coated with a self-antigen) had comparable performance to normalizing by 1 or 3 of the beads with lowest MFI in the assay, independent of locus. The biggest difference in MFI measurements was found between laboratories 1 and 3. When plotting the SAB class I MFIs of these 2 laboratories against each other (Figure 1A), we found lab 3 to consistently measure higher values than lab 1. In Figure 1B, STBR-6 ratio (using bead of the same HLA locus with lowest MFI as background) for the same measurements is depicted. We found that the measurement bias from Figure 1A is not present anymore in the STBR-6 ratio measurements. An effect also clearly expressed by the strongly reduced MARDs for lab 1 versus lab 3 for all the ratios (Tables 2 and 3).

For the class II assay, we did not encounter the same bias between lab 1 and lab 3 (Figure 1C). The MARD between laboratories 1 and 3 for class II (32%, Table 3) was also considerably smaller than that for class I (51%, Table 2). For class II, none of the STBRs had consistently smaller MARDs than the MFI; except STBR-2, the STBRs had a between-laboratories variability comparable to the raw MFI. We therefore conclude that STBRs seem very useful to mitigate proportional bias, while remaining of comparable variability (MARD between 20% and 35%) to that of the raw MFI if no such bias is present.

### Assessment of Impact on Graft Survival

**FIGURE 2.**
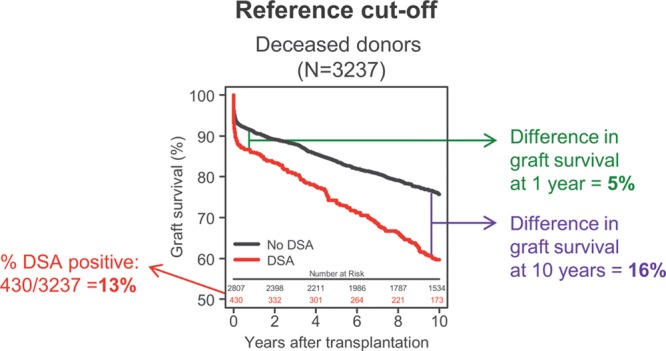
Adjusted Kaplan-Meier death-censored graft survival estimates according to the presence of pretransplant donor-specific HLA antibody (DSA) cutoff determined according to manufacturers’ instructions. The impact of various cutoffs was related to the difference in graft survival of 5% at 1 year (green) and of 16% at 10 years after transplant for 430 (13%) of 3237 DSA-positive transplants.

In a previous study,^3^ we found that the impact of SAB-detected DSA on death censored graft survival, adjusted for differences in other covariables, is most pronounced in transplants with grafts from deceased donors. In this study, bead positivity was determined according to manufacturer instructions. A lot-specific background MFI per bead (range 150–400) was provided, to be subtracted from the raw MFI value (referred to as the BCM value). In addition, 2 ratios were to be calculated: the BCR as the BCM divided by the lowest MFI of all beads with antigens of the same locus, and the AD-BCR as the BCR divided by the relative amount of antigen coated on the bead. When 2 of the 3 values (BCM, BCR, AD-BCR) are above a certain lot-specific threshold, respectively, 1500, 3, and 4 for the lots that we used, the bead is deemed positive. With bead positivity, as suggested by the manufacturer, we found that the 13% of transplants positive for DSA in pretransplant serum have a 5% poorer death-censored and covariable-adjusted graft survival after 1 year and 16% after 10 years (Figure 2).

**FIGURE 3.**
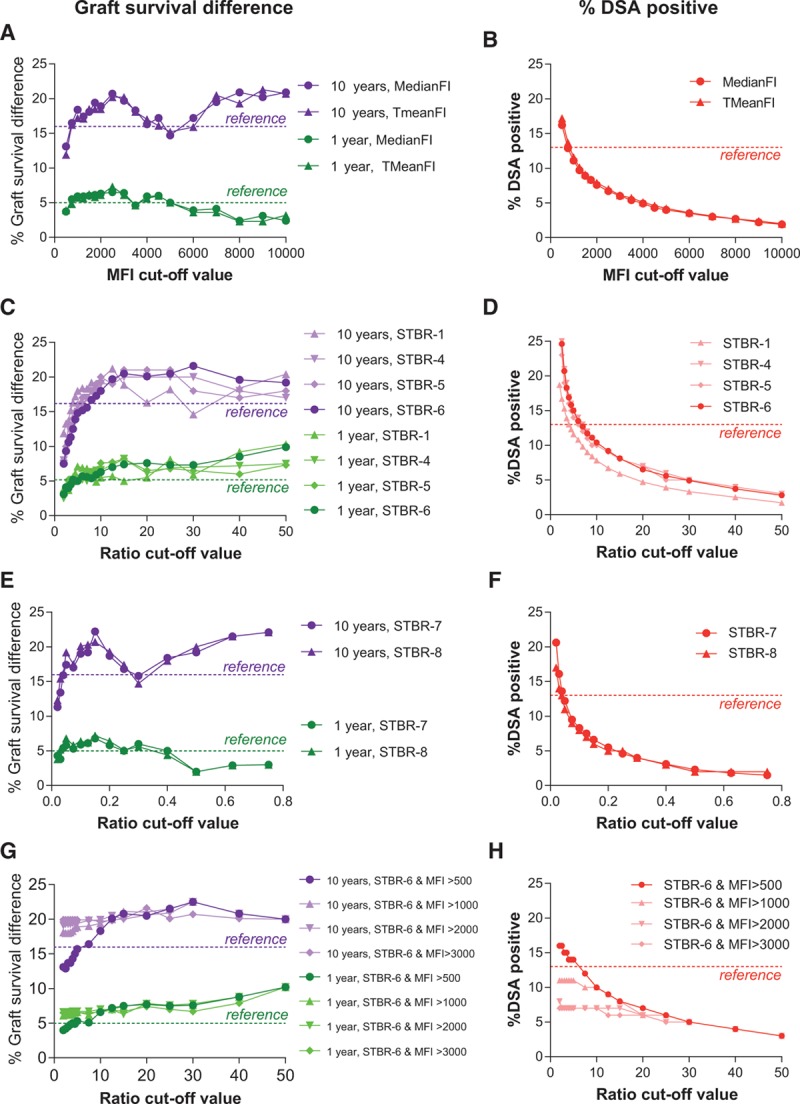
A, Relationship between difference in graft survival 1 (green) and 10 (purple) years after transplant for absolute MFI cutoffs (median and 5%-trimmed mean) with (B) the corresponding percentage of DSA-positive transplants (red). The dotted lines represent the reference cutoff as determined according to manufacturer’s instructions and also depicted in Figure 2: 5% difference in 1-year graft survival (green); 16% difference in 10-year graft survival (purple), and 13% DSA-positive transplants for the manufacturer’s cutoff. C, Relationship between difference in graft survival 1 (green) and 10 (purple) years after transplant for STBR cutoffs: STBR-1 using the negative control beads as background MFI, STBR-4 using the average MFI of the lowest 5-bead measurements as background MFI, STBR-5 using the average MFI of the lowest 10-bead measurements as background MFI, and STBR-6 with background MFI with the bead of the same HLA locus with lowest MFI, with (D) the corresponding percentage of DSA-positive transplants (red). E, Relationship between difference in graft survival 1 (green) and 10 (purple) years after transplant for STBR-7 (using positive control bead) and STBR-8 (using the negative and positive control bead) with (F) the corresponding percentage of DSA-positive transplants (red). G, Relationship between difference in graft survival 1 (green) and 10 (purple) years after transplant for STBR-6 in combination with absolute MFI cutoffs with (H) the corresponding percentage of DSA-positive transplants (red). See Table 1 for a detailed description of the various STBRs with the background used for the calculation. DSA, donor-specific HLA antibody; MFI, median fluorescence intensity; STBR, signal-to-background ratio.

In the current study, we evaluated the impact of different cutoff levels for absolute MFI measurements, a range of STBRs, and combinations thereof on death-censored and covariable-adjusted graft survival. We did not find a strong or even nondecreasing relationship between absolute MFI cutoff and graft survival difference between the resulting DSA-positive and DSA-negative transplants, both when the MFI was evaluated as median and 5% trimmed mean (Figure 3A). The dotted lines in Figure 3 represent the reference cutoff as determined according to the manufacturer’s instructions as depicted in Figure 2: 5% difference in 1-year graft survival (green), 16% difference in 10-year graft survival (purple), and 13% DSA-positive transplants for the manufacturer’s cutoff. A strong increase is visible in both short- and long-term graft survival differences between transplants with and without DSA when the MFI cutoff is increased to a value of 750 to 1000, with a (local) maximum around an MFI of 2500 (based on the previous section, we would like to stress that these values are laboratory specific and do not hold in general). With the cutoff increasing from 2500 to 10 000, the 1-year graft survival difference decreased, however. The 10-year graft survival difference between DSA-positive and -negative transplants did not increase for cutoffs between 2500 and 10 000 either and was actually 5% lower for a cutoff around 5000. The percentages of DSA-positive transplant for each MedianFI or TMeanFI cutoff value are shown in Figure 3B.

A much clearer relationship between cutoff level and graft survival difference was found for the STBRs (Figure 3C), especially with respect to short-term survival. For all STBRs, the 1-year graft survival difference is nondecreasing (STBR-4 and STBR-5) or even increasing (STBR-1 and STBR-6) with increasing cutoff value. The MFI of the bead of the same HLA locus with lowest MFI as background provided the most stable relationship between cutoff and 1-year graft survival; the MFI of the negative control bead as background led to a much less consistent relationship. Whereas for STBR-1, no robust relationship was found either between cutoff value and 10-year graft survival difference, STBR-4, STBR-5, and STBR-6 showed a much more stable relationship. The STBR-4 and STBR-5 ratios showed a decrease in 10-year graft survival difference for higher cutoff values. The 10-year graft survival difference remained much more stable for STBR-6. Of all the STBRs, the bead of the same HLA locus with lowest MFI therefore seems to provide the preferable background signal for risk stratification based on short- and long-term graft survivals.

In Figure 3C, we evaluated MFI measurements relative to background signals we considered to be negative. The SAB kit, however, also contained a positive control bead, coated with human IgG. We next investigated the potential additional value of expressing the MFI measurement of a bead relative to the MFI of this positive control bead. To this end, we calculated 2 additional ratios. The first, STBR-7, was derived as the MFI of a specific bead divided by the MFI of the positive control bead. In the second, STBR-8, the relative position of the MFI of a specific bead was determined between the negative (set to 0) and the positive control bead (set to 1) (Figure 3E). The relationship between cutoff level and graft survival difference was highly comparable to the one found for raw MFI cutoffs (Figure 3A), both, and short- and long-term for both ratios. The MFI of the positive control bead therefore did not seem to be of additional value in the bead positivity assessment.

Background MFIs can be extremely low; for example, we encountered values as low as 2 in our data set. When working with STBRs, such low background signals lead to beads with low MFIs (eg, in the range of 50–100) being assessed as positive, even if a high ratio cutoff level is chosen. We therefore investigated if requiring a minimum MFI level in addition to ratio cutoff would be of additional value. We combined all STBR cutoffs with MFI cutoffs between 500 and 3000. For the STBR-6 ratio cutoff, we again found the clearest and most stable relationships between cutoff level and graft survival difference, displayed in Figure 3D for additional MFI cutoffs of 500, 1000, 2000, and 3000. For smaller STBR-6 ratio cutoff levels, having an additional MFI cutoff resulted in a strong increase in graft survival difference between the DSA-positive and -negative groups and a strong decrease in DSA positivity. Yet the higher the MFI cutoff taken, the more this cutoff will start to dominate in the bead positivity assessment, whereas from Figure 3A and B, it seems clear that a dominant MFI cutoff would not be desirable. We would therefore advise a modest MFI cutoff level of around 500 in addition to an STBR cutoff. For higher STBR cutoff levels (above 10), additional MFI cutoffs do not positively impact short- and long-term graft survival differences anymore and mainly lead to less transplants classified positive for DSA.

This brings us to a final criterion in the cutoff selection that has received only modest attention so far: DSA positivity. We have shown that when working with the STBR-6 ratio, the higher the cutoff value used, the higher the graft survival difference between resulting DSA-positive and -negative transplants. At the same time, the percentage of transplants classified positive for DSA (strongly) decreases of course with increasing the cutoff. Therefore, in choosing an appropriate cutoff level for risk stratification, a balance needs to be sought between impact (in this case graft survival difference) on the one hand, and prevalence (% DSA positive) on the other.

Figure 3B, D, F, and H represent the percentage of DSA-positive transplants for different cutoffs. Which cutoff provides the appropriate balance between impact and prevalence is a managerial problem rather than a mathematical one. Nonetheless, from a mathematical perspective, we can provide the following input: we found the STBR-6 ratio cutoffs (MFI of a specific bead divided by bead of the same HLA locus with lowest MFI) to have the clearest and most stable relationship with short- and long-term graft survival differences. In Figure 3C, we see that from an STBR-6 cutoff of 15 and higher, the graft survival difference is stabilizing (no increase in graft survival difference with decreasing % DSA–positive patients, Figure 3D). To avoid clear false positives, one could consider combining the STBR-6 ratio cutoff with an MFI cutoff. From Figure 3G and H, we can derive that when an STBR-6 cutoff of 15 is combined with an MFI cutoff of 500 or 1000, the same balance between graft survival difference and DSA positivity can be reached while greatly reducing the risk of false positives. In fact, the addition of the absolute MFI cutoff of 500 to an LRA cutoff of 15 resulted in our cohort in the reclassification of 1 transplant from DSA-positive to -negative only (with an MFI of 296 for the bead initially leading to DSA positivity). **Figure S3 (SDC,** http://links.lww.com/TP/B601) provides a comparison of the reference cutoff (based on manufacturers’ recommendation), and the STBR-6 cutoff of 15 is combined with an MFI cutoff of 500.

## DISCUSSION

Different cutoffs for DSA positivity are used in the literature that are not based on actual results but just on the experience in the different HLA laboratories and transplant centers. Previously, we showed that the presence of pretransplant SAB-defined DSA is associated with increased risk of graft failure.^3^ However, risk stratification based on pretransplant DSA can be further improved by selecting an optimal clinically relevant cutoff for DSA positivity based on graft survival results. Inclusion of de novo DSA could also improve risk stratification; unfortunately, we were not able to investigate this. The data presented in this article show the STBR-6 ratio, the MFI of a specific bead divided by the lowest MFI of all beads of the same HLA locus, to be more robust (across different laboratories) and better related to inferior graft survival for DSA-positive patients than absolute MFI measurements. The combination with a low MFI cutoff around 500 avoids false positivity due to exceptionally low MFI measurement of the background signal (lowest beads of the same locus, probably self-antigen). When applying the manufacturer’s instructions for SAB bead positivity assessment, 13% of the deceased-donor transplants in our cohort are regarded as positive for pretransplant DSA, with, respectively, a 5% and 16% lower death-censored and covariable-adjusted graft survival than transplants negative for DSA. For risk stratification, we propose an STBR-6 cutoff of 15 combined with an MFI cutoff around 500. With this combined cutoff, the focus is redirected on 8% of the deceased-donor transplant now positive for DSA, with, respectively, 7% and 21% difference in 1- and 10-year graft survivals.

All results discussed are based on assays performed with SAB kits of 1 manufacturer and cannot be extrapolated to kits of another manufacturer. However, as the STBR-6 uses the bead of the same HLA locus with lowest MFI as background, this calculation could also be performed for the other vendor’s kit. We evaluated sera from just before the transplant, not (necessarily) peak sera. The results we would have obtained using peak sera might have been different. We acknowledge that antibody-mediated rejection (AMR) would have been a far better criterion to assess clinical relevance of SAB measurements than the eventual graft survival, which is influenced by so many other important factors. Unfortunately, AMR information was not available in this retrospective study. Another limitation of the study is that the sera were prescreened with the Lifescreen deluxe assay, and only the positive sera were tested with the SAB assay. Using this strategy, quite weak DSA might have been missed as the screening assay has a lower sensitivity. Finally, as the presence of DSA was assigned using serological spilt-level donor HLA typing, it is possible that some HLA antibodies are in fact not donor-specific.

In our previous study,^3^ we did not observe an effect on the number of DSAs on graft survival; therefore, we did not further analyze what the impact on graft survival and the number of DSAs would be using a different cutoff strategy. As the combination of DSA class I and/or II was already relatively low using the manufacturer’s cutoff, we did not further investigate the different STBRs because the numbers will get even lower with increasing cutoff. In this study, we only evaluated equal cutoff for both SAB class I and II assays. The effect of different MFI cutoffs and STBR-6 cutoffs is shown in **Figure S1 and Figure S2** (SDC, http://links.lww.com/TP/B601), respectively. From these results, it seems that using a higher MFI cutoff for class II compared with class I appeared more optimal, as higher cutoff showed larger graft survival difference (**Figure S1A and B, SDC,** http://links.lww.com/TP/B601; the above diagonal graft survival differences are considerably higher than those below the diagonal). However, none of the combinations of different MFI cutoffs for class I and II had a better performance than the combined STBR-T MFI cutoff we propose above. Furthermore, the STBR-6 ratios do not suffer from asymmetry in results when different cutoffs are used for SAB class I and class II assays (**Figure S2A and B, SDC,** http://links.lww.com/TP/B601).

We noticed for both MFI measurements and investigated ratios a relatively limited impact of the cutoff level on the decreased graft survival of DSA-positive transplants. When leaving the lower range of cutoffs out of consideration, the 1-year graft survival difference fluctuates around 7%, and the difference 10 years after transplant is around 20%. If the cutoff level would be positively related to the amount of specific antibody present in the tested serum, then it appears that mainly the presence of DSA would be of influence on graft survival rather than the amount. On the other hand, we did observe a weak relationship between cutoff level and 1-year graft survival difference for the STBR-6 ratio, a relationship that might have shown to be stronger if we would have been able to look at AMR instead of graft survival.

In conclusion, in this study, we show that the interassay variability can be greatly reduced and stabilized to an average absolute relative difference between 20% and 35%, when working with STBR instead of absolute MFIs. This normalization makes it possible to choose a uniform STBR cutoff that can be used across different laboratories. Application of the STBRs also resulted in the clearest and most stable relationship between DSA positivity and inferior 1- and 10-year covariable-adjusted graft survivals. With respect to risk stratification of short- and long-term graft survivals, we propose an STBR based on STBR-6 with a cutoff level of 15 combined with an MFI cutoff around 500. For this cutoff, the 1- and 10-year graft survivals are 8% and 21% poorer, respectively, for the 8% of the transplants classified positive for DSA.

## Supplementary Material
