# Supplementary material for: Toward a Sensible Single-antigen Bead Cutoff Based on Kidney Graft Survival
Source: Transplantation. 2019 Mar 22;103(4):789–97. doi: 10.1097/TP.0000000000002357 (PMC6430591; doi:10.1097/TP.0000000000002357)
Supplement: Supplementary file 1 [file tp-103-789-s001.pdf]

Figure S1: Effect of different MFI cut-offs for SAB class I and class II assay

a. Death censored covariable adjusted 1-year graft survival difference between transplantations with and without pretransplant DSA

Class I MFI cut-off \ Class II MFI cut-off	500	750	1000	2000	3000	4000	5000	10000
500	3,7%	4,6%	5,0%	5,3%	5,9%	6,1%	6,2%	6,0%
750	4,4%	5,5%	6,0%	6,4%	7,0%	7,4%	7,3%	6,8%
1000	4,3%	5,4%	5,9%	6,0%	6,7%	7,2%	7,2%	6,7%
2000	4,0%	5,7%	6,4%	6,3%	7,3%	7,7%	7,8%	7,6%
3000	4,1%	5,5%	5,7%	5,5%	6,4%	7,0%	7,1%	6,0%
4000	3,6%	4,4%	4,6%	4,1%	5,0%	5,9%	6,1%	4,3%
5000	3,0%	3,7%	3,8%	3,1%	3,8%	4,7%	5,0%	2,6%
10000	2,7%	3,4%	3,3%	2,8%	3,8%	5,0%	5,4%	2,4%

b. Death censored covariable adjusted 10-year graft survival difference between transplantations with and without pretransplant DSA

Class I MFI cut-off \ Class II MFI cut-off	500	750	1000	2000	3000	4000	5000	10000
500	13,1%	15,4%	16,9%	16,8%	16,3%	15,4%	15,6%	16,5%
750	13,8%	16,5%	18,1%	17,8%	17,5%	16,5%	16,6%	17,4%
1000	13,9%	16,7%	18,4%	18,1%	17,7%	16,2%	16,5%	17,3%
2000	13,7%	16,9%	19,1%	18,9%	19,0%	17,4%	18,0%	19,5%
3000	13,9%	17,4%	19,5%	19,4%	19,7%	18,3%	19,1%	21,9%
4000	13,0%	16,1%	18,3%	17,6%	18,1%	16,3%	17,3%	19,7%
5000	11,7%	14,7%	16,8%	15,8%	15,6%	13,3%	14,7%	16,8%
10000	11,2%	14,6%	16,9%	16,4%	17,2%	13,6%	15,4%	20,9%

c. Percentage of transplantations positive for DSA

Class I MFI cut-off \ Class II MFI cut-off	500	750	1000	2000	3000	4000	5000	10000
500	16,2%	14,5%	13,3%	12,1%	11,5%	11,1%	10,8%	10,0%
750	14,8%	12,9%	11,7%	10,4%	9,7%	9,4%	9,0%	8,2%
1000	14,1%	12,3%	11,1%	9,7%	9,0%	8,6%	8,2%	7,4%
2000	12,5%	10,4%	9,2%	7,6%	6,7%	6,2%	5,8%	4,9%
3000	11,9%	9,8%	8,5%	6,9%	6,0%	5,4%	5,1%	4,0%
4000	11,5%	9,3%	8,0%	6,4%	5,5%	4,9%	4,5%	3,5%
5000	11,0%	8,8%	7,6%	5,9%	5,0%	4,4%	4,0%	2,9%
10000	10,3%	8,1%	6,8%	5,1%	4,1%	3,4%	3,0%	1,9%

Figure S2. Effect of different STBR-6 cut-offs for SAB class I and class II assays

A. Death censored covariable adjusted 1-year graft survival difference between transplantations with and without pretransplant DSA

Class II STBR-6 cut-off

Class I STBR-6 cut-off

Classs	2,5	5	7	10	15	20	30	40	50
2,5	4,1%	4,8%	5,1%	6,0%	6,3%	6,0%	6,2%	6,0%	6,4%
5	4,9%	5,0%	5,4%	6,5%	6,7%	6,7%	7,0%	7,0%	7,6%
7	5,1%	5,1%	5,6%	6,8%	7,1%	6,7%	7,1%	7,1%	7,8%
10	5,2%	5,0%	5,3%	6,3%	6,1%	5,5%	5,6%	5,7%	6,4%
15	5,2%	5,4%	6,0%	7,4%	7,4%	6,9%	7,1%	7,7%	8,7%
20	5,4%	5,9%	6,3%	7,9%	8,0%	7,6%	8,0%	8,8%	10,2%
30	5,5%	5,4%	5,7%	6,9%	7,4%	6,9%	7,3%	8,2%	10,3%
40	5,5%	5,1%	5,3%	6,5%	7,1%	6,7%	7,4%	8,5%	11,0%
50	5,2%	4,7%	5,0%	6,1%	6,8%	5,3%	6,0%	7,1%	9,9%

B. Death censored covariable adjusted 10-year graft survival difference between transplantations with and without pretransplant DSA

Class II STBR-6 cut-off

Class I STBR-6 cut-off

	2,5	5	7	10	15	20	30	40	50
2,5	9,3%	12,1%	12,5%	13,8%	13,5%	13,4%	13,3%	12,9%	12,2%
5	11,1%	14,8%	15,9%	17,6%	17,5%	17,2%	17,3%	16,2%	15,5%
7	11,0%	14,6%	15,6%	17,5%	17,4%	16,4%	16,5%	15,3%	14,3%
10	11,4%	15,0%	16,1%	18,0%	17,5%	16,2%	16,0%	14,9%	13,6%
15	11,7%	16,5%	18,5%	21,1%	20,5%	19,5%	20,2%	19,1%	17,6%
20	11,1%	16,2%	18,1%	21,4%	20,8%	20,1%	21,4%	20,2%	18,3%
30	11,4%	16,1%	18,1%	21,3%	20,5%	19,9%	21,6%	19,4%	17,7%
40	10,8%	15,2%	17,3%	20,5%	19,8%	19,4%	21,9%	19,6%	18,1%
50	10,6%	15,2%	17,4%	21,0%	20,4%	19,7%	23,0%	20,6%	19,2%

C. Percentage of transplantations positive for DSA

Class II STBR-6 cut-off

Class I STBR-6 cut-off

	2,5	5	7	10	15	20	30	40	50
2,5	24,6%	19,0%	17,7%	16,7%	16,0%	15,7%	15,0%	14,7%	14,4%
5	21,4%	15,0%	13,5%	12,4%	11,6%	11,0%	10,2%	9,8%	9,5%
7	20,9%	14,2%	12,7%	11,6%	10,8%	10,2%	9,3%	8,9%	8,6%
10	20,0%	13,1%	11,5%	10,4%	9,5%	8,9%	8,0%	7,5%	7,2%
15	19,1%	11,9%	10,2%	9,1%	8,1%	7,4%	6,4%	5,9%	5,6%
20	18,4%	11,2%	9,5%	8,3%	7,3%	6,5%	5,6%	5,1%	4,7%
30	18,0%	10,7%	9,0%	7,7%	6,6%	5,9%	4,9%	4,4%	4,0%
40	17,6%	10,1%	8,4%	7,2%	6,0%	5,3%	4,2%	3,7%	3,3%
50	17,2%	9,7%	8,0%	6,8%	5,7%	4,8%	3,8%	3,2%	2,8%

Figure S3. Adjusted Kaplan-Meier estimates (AKME) for death-censored graft survival according to the presence of pretransplant DSA for the total cohort using the cut-off as recommended by the manufacturer as a reference or the STBR6 cut-off of 15 combined with an MFI cut-off of 500.

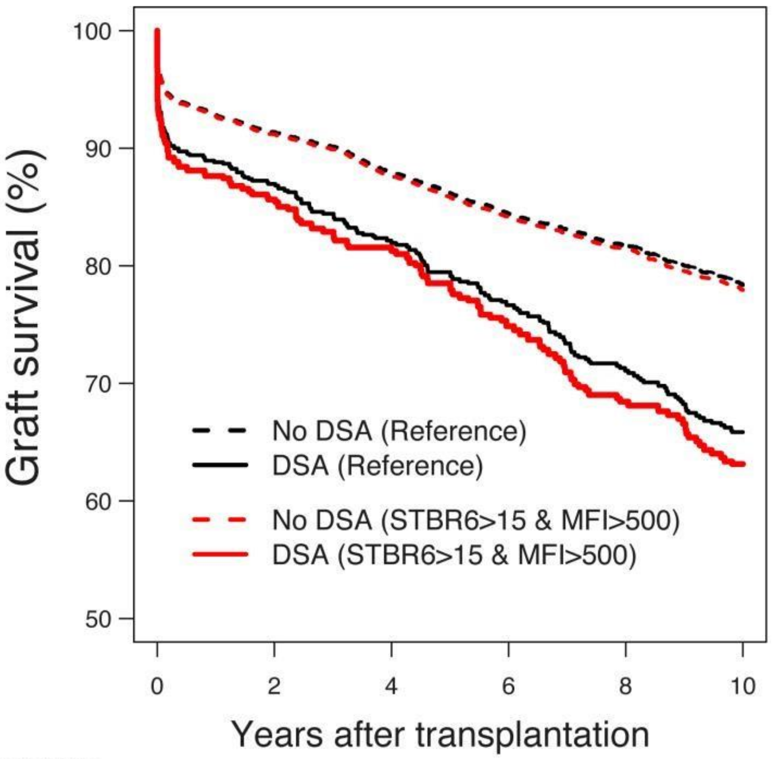

Number at Risk						
No DSA (Reference)	4157	3665	3425	3122	2844	2461
DSA (Reference)	567	457	420	373	318	259
No DSA (STBR6>15 & MFI>500)	4373	3846	3592	3274	2976	2576
DSA (STBR6>15 & MFI>500)	351	276	253	221	186	144
